# Gut microbial features may influence antiviral IgG levels after vaccination against viral respiratory infectious diseases: the evidence from two-sample bidirectional mendelian randomization

**DOI:** 10.1186/s12879-024-09189-0

**Published:** 2024-04-23

**Authors:** Junlan Tu, Yidi Wang, Xiangyu Ye, Yifan Wang, Yixin Zou, Linna Jia, Sheng Yang, Rongbin Yu, Wei Liu, Peng Huang

**Affiliations:** 1 Department of Epidemiology, Center for Global Health, School of Public Health, National Vaccine Innovation Platform, Nanjing Medical University, 211166 Nanjing, China; 2 Department of Infectious Disease, Jurong Hospital Affiliated to Jiangsu University, Jurong, China; 3 Department of Biostatistics, Center for Global Health, School of Public Health, National Vaccine Innovation Platform, Nanjing Medical University, 211166 Nanjing, China; 4 Beijing Institute of Microbiology and Epidemiology, State Key Laboratory of Pathogen and Biosecurity, 100071 Beijing, China

**Keywords:** Gut microbial features, Immunoglobulin G, Respiratory infectious disease, Two-sample mendelian randomization analysis, Immunity

## Abstract

**Background:**

Vaccination is effective in preventing viral respiratory infectious diseases through protective antibodies and the gut microbiome has been proven to regulate human immunity. This study explores the causal correlations between gut microbial features and serum-specific antiviral immunoglobulin G (IgG) levels.

**Methods:**

We conduct a two-sample bidirectional Mendelian randomization (MR) analysis using genome-wide association study (GWAS) summary data to explore the causal relationships between 412 gut microbial features and four antiviral IgG (for influenza A, measles, rubella, and mumps) levels. To make the results more reliable, we used four robust methods and performed comprehensive sensitivity analyses.

**Results:**

The MR analyses revealed 26, 13, 20, and 18 causal associations of the gut microbial features influencing four IgG levels separately. ​Interestingly, ten microbial features, like genus *Collinsella*, species *Bifidobacterium longum*, and the biosynthesis of L-alanine have shown the capacity to regulate multiple IgG levels with consistent direction (rise or fall). The ​reverse MR analysis suggested several potential causal associations of IgG levels affecting microbial features.

**Conclusions:**

The human immune response against viral respiratory infectious diseases could be modulated by changing the abundance of gut microbes, which provided new approaches for the intervention of viral respiratory infections.

**Supplementary Information:**

The online version contains supplementary material available at 10.1186/s12879-024-09189-0.

## Introduction

Respiratory infectious diseases are caused by pathogens like viruses, bacteria, mycoplasma, and chlamydia through respiratory secretions [1]. Pandemics of respiratory infectious diseases often affect many countries or regions and cause large numbers of deaths [2, 3]. Prevention of respiratory infectious diseases continues to be an important public health initiative and vaccines play a critical role in prevention [4]. However, the capacity of protection by vaccination varies among individuals [5, 6]. Vaccines mediate protection by inducing B cells which produce antigen-specific antibodies [7]. When the host contracts a virus or receives a virus-specific vaccine, Immunoglobulin G (IgG) antibodies are secreted from B cells and bind to a variety of pathogens or antigens to avoid infection or provide protection [8, 9]. Previous studies showed that IgG level strongly correlated with the immune response to vaccination, and the IgG level could reflect the protective function of the specific vaccine [10, 11]. Therefore, finding and intervening factors that affect individual IgG levels can improve vaccine protection function and enhance herd immunity.

Several studies have illuminated important roles for the gut microbiota in modulating B cells response that perhaps have important implications for the effects of the microbiota on specific IgG levels [12]. Moreover, the gut microbiota also produces a large number of metabolites that have the potential to adjust immune responses. Short-chain fatty acids (SCFAs) had been shown to increase acetyl-coenzyme A and regulate metabolic sensors to increase oxidative phosphorylation, glycolysis, and fatty acid synthesis in B cells to support antibody production, and had been shown to enhance the expression of genes involved in plasma cells (effector B cell) differentiation [7]. In addition, gut microbiota could be regulated in a convenient and harmless way through daily diet, probiotics, and prebiotics [13]. Thus, it is necessary to study the specific IgG level of immune response to vaccination against respiratory infectious diseases from the perspective of gut microbiota.

Influenza A, measles, rubella, and mumps caused by viruses are typical respiratory infectious diseases of concern worldwide. In European, the vaccination rates for the four infectious diseases were high, which contributed to the high seroprevalences of these four virus-specific IgG levels [14]. Based on the high seroprevalences, this research had enough sample sizes to explore the causal correlation between gut microbiota and virus-specific IgG levels.

Mendelian randomization (MR), regarding genetic variants as instrumental variables (IVs) to explore the causal correlations between risk factors and diseases, has been widely used in causal inference [15]. Two-sample MR is an MR-based research method applicable to situations where exposure and outcomes are derived from two different populations [16]. Here, we used two-sample MR to illustrate the causal relationships between gut microbiota and serum virus-specific IgG levels of viral respiratory infectious diseases.

## Methods

### Data collection and processing

The genome-wide association studies (GWAS) summary statistics of 412 gut microbial features (including 207 microbial taxa and 205 functional pathways) were collected from NHGRI-EBI GWAS Catalog (https://www.ebi.ac.uk/gwas/downloads/summary-statistics), which originally came from the *Dutch Microbiome Project* (DMP), a sub-project of *LifeLines* in Netherlands, and it investigated feces and phenotype information to assess the impact of different exposures and lifestyles on gut microbial composition using 7738 *LifeLines* participants [17, 18]. Of these DMP participants, 58.1% were female, mean age was 48.5 years, and mean BMI value was 25.58.

Specifically, the gut microbial taxa were divided according to Taxonomy, using “s_”, “g_”, “f_”, “o_”, “c_”, “p_”, and “k_” to represent species, genus, family, order, class, phylum, and kingdom, respectively. The pathways were identified from the MetaCyc Metabolic Pathways Database (https://metacyc.org/ ), presenting in gut microbes and involving primary and secondary metabolism, as well as associated metabolites, reactions, enzymes, and genes [19]. ​Due to the lengthy names of functional pathways, we chose to use specific abbreviations, the full names of which were given in Supplementary Table S1. We regarded standard deviation (*s.d.*) as the unit of change of gut microbial features (Supplementary Table S2).

In addition, we estimated the heritability (*h*^*2*^) of all 412 gut microbiome features using linkage disequilibrium score regression (*LDSC*) (v1.0.1), with the 503 European individuals from the 1000 Genome Project as the reference panel for analysis [20].

We collected four virus-specific immunoglobulins G (IgG) levels datasets (anti-IAV IgG, anti-measles virus IgG, anti-rubella virus IgG, and anti-mumps virus IgG levels) focusing on long-term immunity also from the NHGRI-EBI GWAS Catalog, and the datasets originally were from 1,000 healthy individuals of the *Milieu Interieur* (MI) cohort in France [21]. This cohort consisted of 500 males and 500 females, with an age range between 20 and 70 years old. For the four mentioned IgG levels, Elisa or multiplex EIA techniques were used for quantitative detection, and the seroprevalence rate were 77.7%, 88.5%, 93.5%, and 91.2% separately [14]. ​We also used *s.d.* as the unit of change.

Then, we filtered the single nucleotide polymorphisms (SNPs) in the abovementioned 416 data for subsequent analyses [22, 23]. We retained the SNPs following: (i) on the autosomes (1–22); (ii) minimum allele frequency (MAF) larger than 0.001; (iii) more than 70% of observers had these specific SNPs.

### MR analysis

**Fig. 1:**
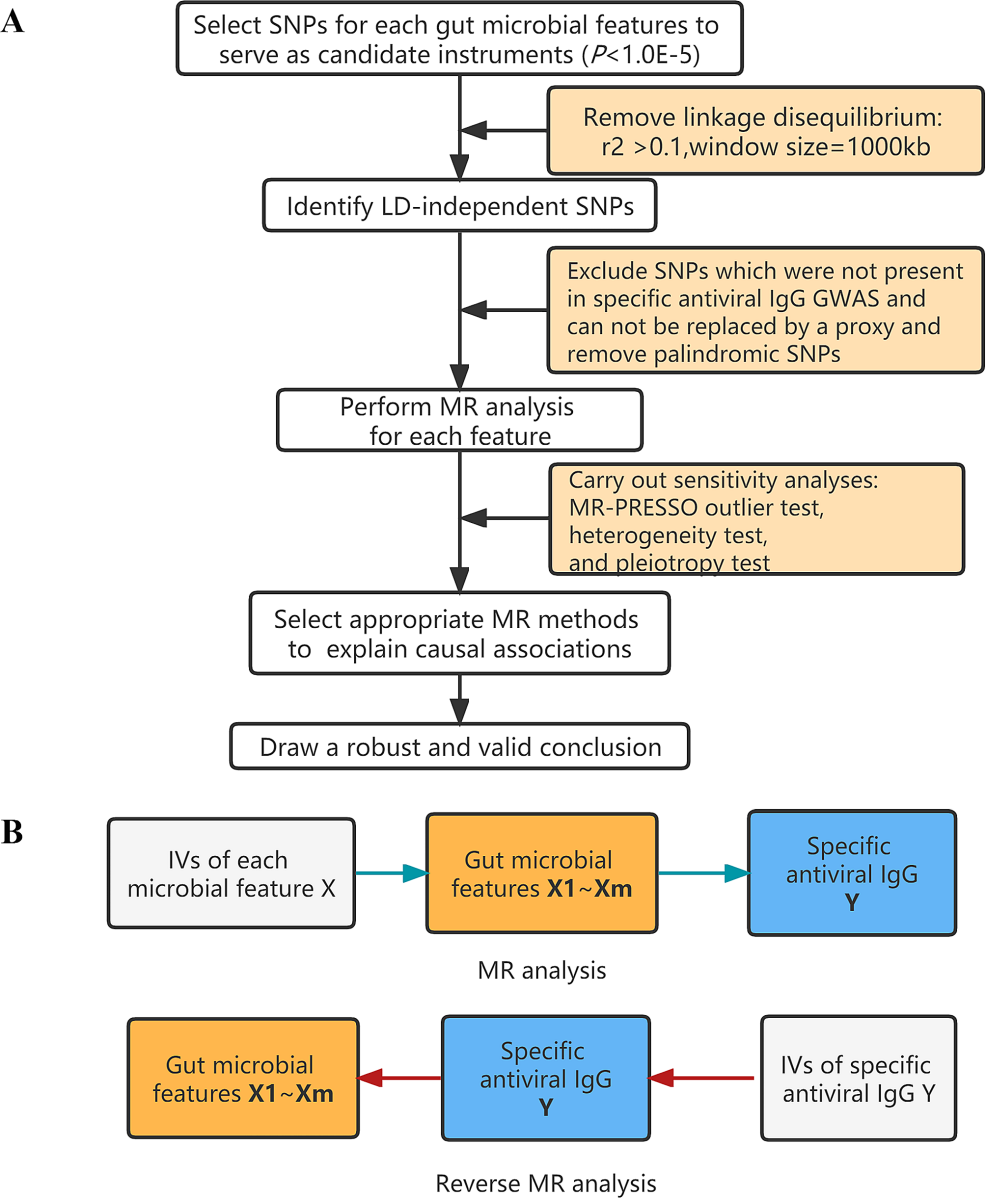
The workflow diagram of this study. Plot **A** lists the detailed steps of two-sample MR in this study mentioned in the method, and Plot **B** shows how to do two-sample MR bidirectionally. X represents one of 412 microbial features, while Y represents one of 4 IgG levels

Two-sample MR is a method to estimate the causal effect of an exposure on an outcome using only summary statistics from GWAS [24, 25]. We treated the gut microbial features as exposures and the four anti-virus IgG levels as outcomes to conduct a two-sample MR study. Based on the datasets, we followed a strict screening procedure to select IVs in other previous MR studies [26, 27] (Fig. 1). First, we used the suggestive threshold *P* < 1E-5 to select genetic variations associated with each particular gut microbial feature. Second, we performed the clumping process [linkage disequilibrium (LD) r^2^ < 0.1, sliding window of 1 Mb] in the reference panel and retained identified LD-independent SNPs. Third, for each respiratory infectious disease, we screened the GWAS database to exclude SNPs associated with a specific IgG level to avoid potential pleiotropy and removed palindromic SNPs. Specifically, if the number of IVs was three or less, we excluded the microbial feature.

To explore the potential causal effects of gut microbial features on the four IgG levels, we performed two-sample MR analyses using the four methods including fixed-effects (IVW (fe)) and multiplicative random-effects inverse variance weighting (IVW (mre)), weighted median (WM), and MR-Egger regression methods in the *TwoSampleMR* (v0.5.6) R package [28–30]. Importantly, we prefer to interpret the results based on the IVW (fe) model without heterogeneity or pleiotropy of IVs [31]. The MR-Egger regression model, where its intercept was used to evaluate the directional pleiotropy of instruments, is preferred in the presence of pleiotropy while the WM method is preferred to account for it in the presence of heterogeneity [32, 33]. As a result, IVW (fe) and MR-Egger regression methods were mainly used to estimate their causal effects with *P* < 0.05. We also performed false discovery rate (FDR) to adjust the false positive rate.

### Sensitivity analysis

First, we used Cochran’s Q test to conduct a heterogeneity test to examine the differences between IVs. We used the *P* value of Q statistics < 0.05 as the significant level. Second, we performed a pleiotropy test. When there was a statistical difference between the intercept and zero (*P* < 0.05), horizontal pleiotropy existed. In addition, we performed Mendelian Randomization Pleiotropy RESidual Sum and Outlier analysis (MR-PRESSO) to detect and correct the effects from outliers [34].

### Reverse-direction MR analysis

We were also concerned about whether these four IgG levels affect the abundance of gut microbial features in humans. We used the same settings as the above-mentioned MR analysis (*P* = 1.0E-5, r^2^ = 0.1, and window size = 1 Mb) to select IVs of four antiviral IgG levels (Fig. 1). In addition, we computed the sum of values of variance in phenotype explained (PVE) to assess the explanatory power of IVs, and computed the *F* statistics following reference to judge whether IVs are strong instruments [35].

## Results

### Heritability of gut microbial features

**Fig. 2:**
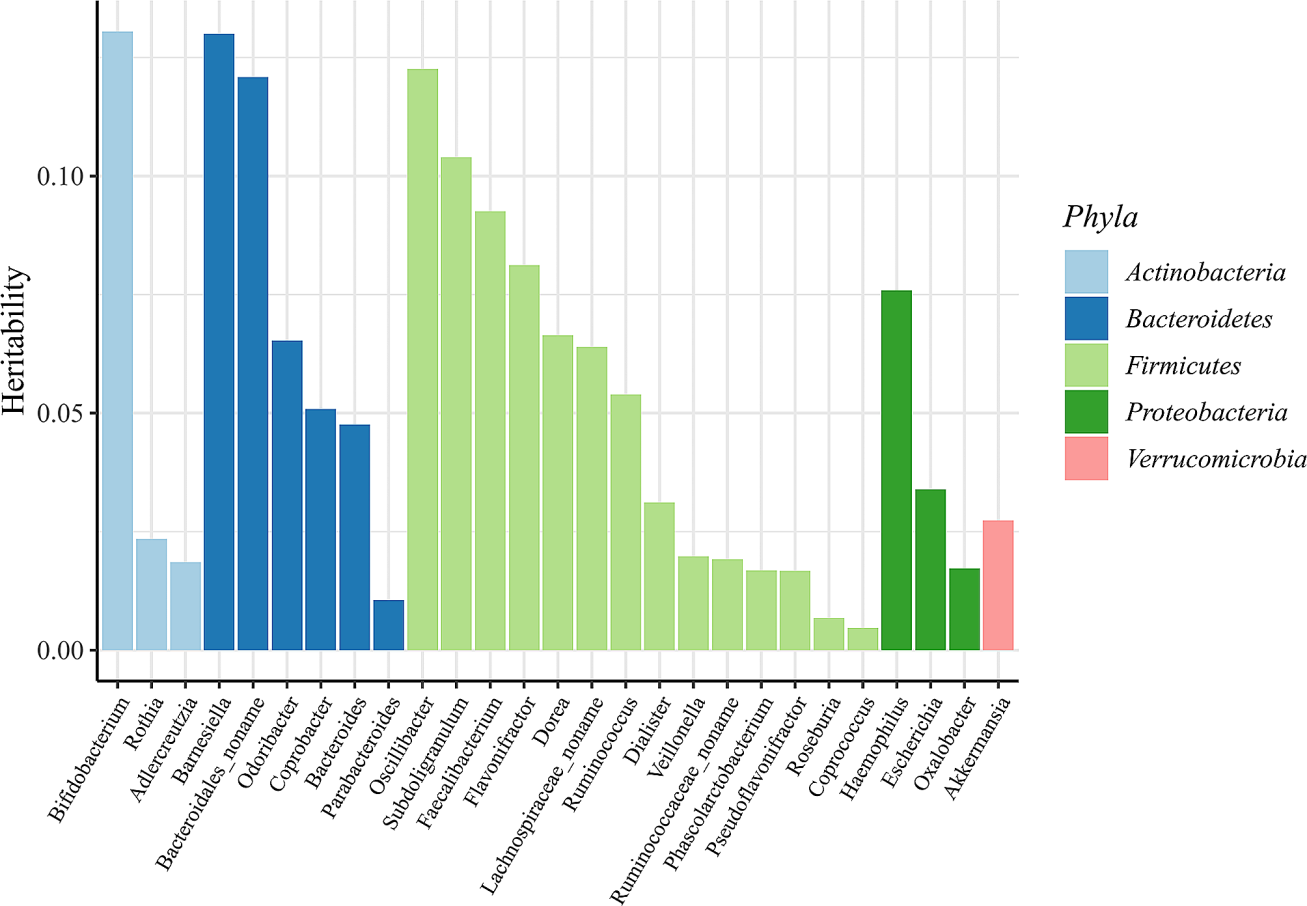
Heritability (*h*^2^) of each microbial feature. The bar chart shows the independent genetic variation for 27 common genera explaining their phenotypic variation. Genera are classified by their 5 respective phyla marked in different colors

Heritability is the proportion of variation in a given gut microbial feature that can be attributed to genetic factors. For all 412 gut microbial features, the median heritability of all features was 5.25%, for example, 22.29% of species *Alistipes senegalensis* and 19.48% of *Aspartate superpathway*, while only 0.02% of family *Prevotellaceae* (Supplementary Table S1). The relative abundance variance of five genera could be explained over 10.00% by their corresponding independent genetic variants, including genus *Bifidobacterium* (13.05%), *Barnesiella* (13.00%), *Bacteroidales noname* (12.08%), *Oscillibacter* (12.26%) and *Subdoligranulum* (10.39%) (Fig. 2).

### MR analyses

We calculated the causal effects of the remained various gut microbial features with four anti-virus IgG levels respectively.

#### Anti-IAV IgG level

It was shown that diverse microbial taxa or functional pathways have different effects on a particular IgG, which manifested as positive or negative causal associations with different effect values. The number of gut microbial features causally associated with anti-IAV IgG level was the largest including 12 taxa and 14 pathways (*P* < 0.05). The taxa all belonged to the four phyla of *Actinobacteria*, *Bacteroidetes*, *Firmicutes*, and *Proteobacteria*, which were consistent with the fact that these four phyla were the largest of the nine identified phyla of human gut microbiota [36].

**Fig. 3:**
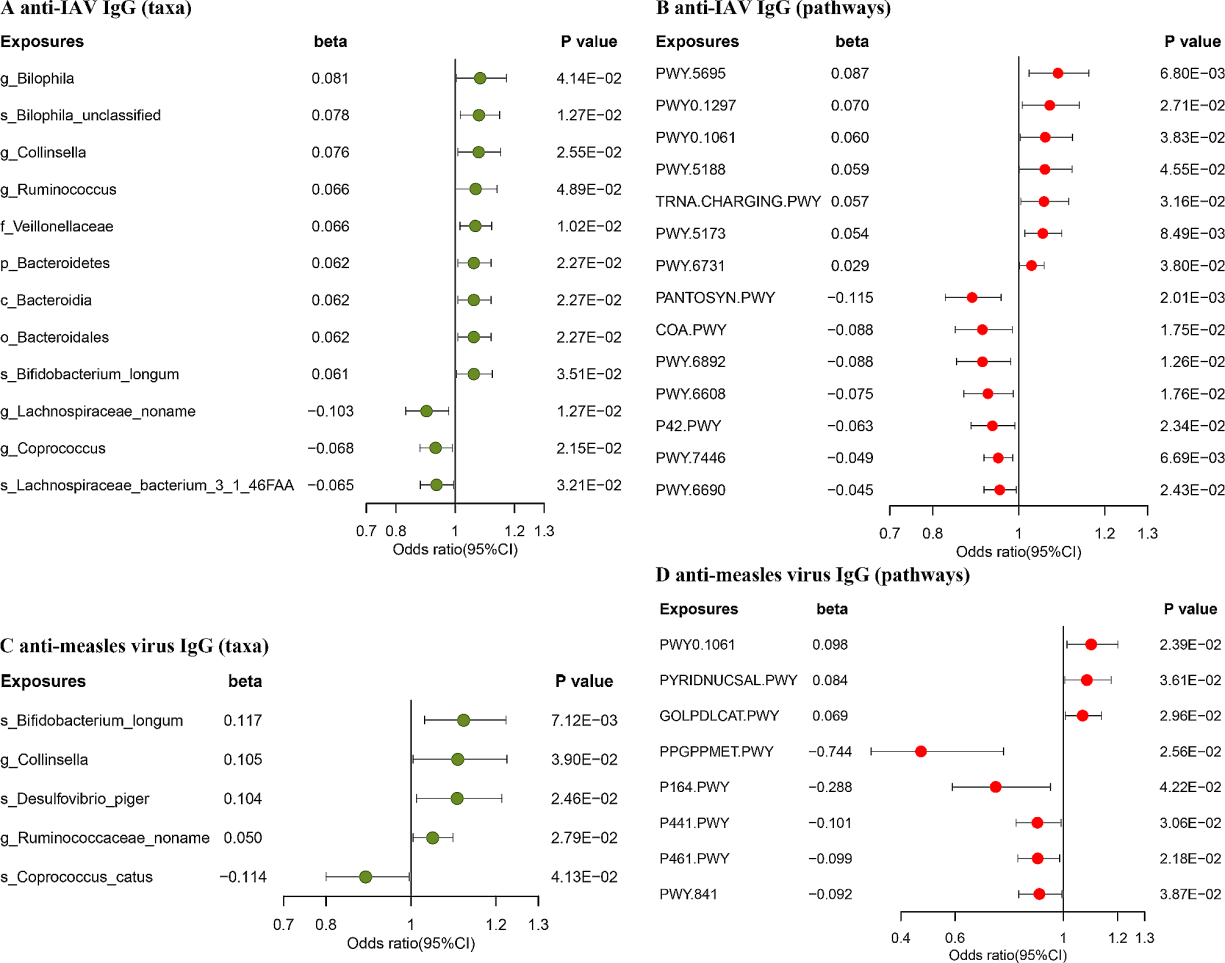
Causal effects of gut microbial features on anti-IAV IgG and anti-measles virus IgG levels. The forest plots represent the MR estimates beta and 95%CI values of the odds ratio of gut microbial features on different serum anti-IAV IgG (**A**, **B**) and anti-measles virus IgG levels (**C**, **D**), as estimated using the fixed effect (IVW) two-sample MR or MR-Egger methods

Of these 12 microbial taxa, nine had the potential to elevate serum anti-IAV IgG concentration, including genus *Bilophila* ($\beta$=0.081, *P* = 0.04), species *Bilophila unclassified* ($\beta$=0.078, *P* = 0.013), genus *Collinsella* ($\beta$=0.076, *P* = 0.026), genus *Ruminococcus* ($\beta$=0.066, *P* = 0.049), family *Veillonellaceae* ($\beta$=0.066, *P* = 0.010), species *Bifidobacterium longum* ($\beta$=0.061, *P* = 0.035), phylum *Bacteroidetes* ($\beta$=0.062, *P* = 0.023), class *Bacteroidia* ($\beta$=0.062, *P* = 0.023), and order *Bacteroidales* ($\beta$=0.062, *P* = 0.023). The results of the last three taxa were similar perhaps because of their affiliations. While three other taxa may decrease anti-IAV IgG level, including genus *Lachnospiraceae noname* ($\beta$=-0.103, *P* = 0.013), genus *Coprococcus* ($\beta$=-0.068, *P* = 0.022), and species *Lachnospiraceae bacterium 3_1_46FAA* ($\beta$=-0.065, *P* = 0.035) (Fig. 3A; Supplementary Table S3).

Similar to taxa, seven pathways had positive causal associations with anti-IAV IgG level, for example, the higher functional capacity for inosine 5’-phosphate degradation (PWY-5695) contributed to a higher level of anti-IAV IgG ($\beta$=0.087, *P* = 0.007). Nevertheless, an increase functional capacity of seven pathways may lower anti-IAV IgG, such as the capacity of pantothenate and coenzyme A biosynthesis (PANTOSYN-PWY, $\beta$=-0.115, *P* = 0.020) with the largest negative effect (Fig. 3B; Supplementary Table S3).

#### Anti-measles virus IgG

The number of gut microbial features causally associated with anti-measles IgG level was 13, involving five taxa and eight pathways (Fig. 3, C-D; Supplementary Table S4). Not entirely consistent with anti-IAV IgG level, these five taxa did not belong to phylum *Bacteroidetes*. Four taxa including species *Bifidobacterium longum* ($\beta$=0.117, *P* = 0.007), genus *Collinsella* ($\beta$=0.105, *P* = 0.039), species *Desulfovibrio piger* ($\beta$=0.104, *P* = 0.025), and genus *Ruminococcaceae noname* ($\beta$=0.050, *P* = 0.028) had the potential to increase anti-measles virus IgG level. Only species *Coprococcus catus* may decrease the titer of anti-measles virus IgG ($\beta$=-0.114, *P* = 0.041).

For pathways, the higher functional capacity for L-alanine biosynthesis (PWY0-1061, $\beta$=0.098, *P* = 0.024), NAD salvage (PYRIDNUCSAL-PWY, $\beta$=0.084, *P* = 0.036), and glycerol degradation to 1,3-propanediol (GOLPDLCAT-PWY, $\beta$=0.069, *P* = 0.030) may elevate the level of anti-measles virus IgG. In addition, an increase functional capacity of five pathways could lower anti-measles virus IgG including ppGpp biosynthesis (PPGPPMET-PWY, $\beta$=-0.744, *P* = 0.026), purine nucleobases degradation (P164-PWY, $\beta$=-0.288, *P* = 0.042), N-acetylneuraminate degradation (P441-PWY, $\beta$=-0.101, *P* = 0.031), hexitol fermentation to hexitol fermentation to lactate, formate, ethanol and acetate (P461-PWY, $\beta$=-0.099, *P* = 0.022), and purine nucleotides de novo biosynthesis (PWY-841, $\beta$=-0.092, *P* = 0.022). Considering the pleiotropy of IVs, the causal effects of P164-PWY (*P* = 0.031) and PPGPPMET-PWY (*P* = 0.026) with anti-measles virus IgG were explained by the results of MR-Egger regression method.

#### Anti-rubella virus IgG

**Fig. 4:**
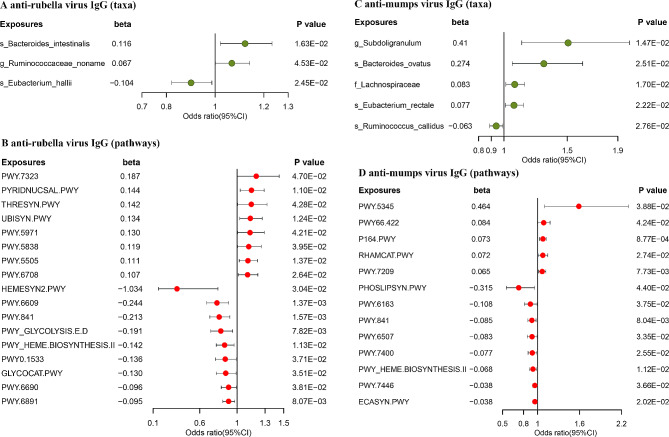
Causal effects of gut microbial features on anti-rubella virus IgG and anti-mumps virus IgG levels. The forest plots represent the MR estimates beta and 95%CI values of the OR of gut microbial features on anti-rubella virus IgG (**A**, **B**) and anti-mumps virus IgG levels (**C**, **D**), as estimated using the fixed effect (IVW) or MR-Egger method

MR analyses showed that three microbial taxa and 17 functional pathways had the potential to influence the anti-rubella virus IgG level (Fig. 4, A-B; Supplementary Table S5). These taxa only involving phylum *Firmicutes* and *Bacteroidetes*. The species *Bacteroides intestinalis* ($\beta$=0.116, *P* = 0.016) and genus *Ruminococcaceae noname* ($\beta$=0.067, *P* = 0.045) perhaps had a positive impact on increasing anti-rubella virus IgG level while the species *Eubacterium hallii* ($\beta$=-0.104, *P* = 0.024) had a negative impact on this IgG level.

The pathways positively correlated with anti-rubella virus IgG were almost biosynthesis pathways, such as L-threonine biosynthesis (THRESYN-PWY,$\beta$=0.142, *P* = 0.043), palmitate biosynthesis (PWY-5971,$\beta$=0.130, *P* = 0.042), and L-glutamate and L-glutamine biosynthesis (PWY-5505,$\beta$=0.111, *P* = 0.014). An increase functional capacity of other eight pathways may decrease anti-measles virus IgG level mainly about biosynthesis or degradation pathways, like purine nucleotides de novo biosynthesis (PWY-841,$\beta$=-0.213, *P* = 0.002), and glycogen degradation (GLYCOCAT-PWY,$\beta$=-0.130, *P* = 0.035). Given that there was pleiotropy of IVs (*P* = 0.048), we used MR-Egger regression method to show the causal effect between the capacity of a kind of heme biosynthesis (HEMESYN2-PWY) and the anti-rubella virus IgG level.

#### Anti-mumps virus IgG

Five taxa and 13 functional pathways may adjust anti-mumps virus IgG (Fig. 4, C-D; Supplementary Table S6). Similar to anti-rubella virus IgG, these taxa also belonged to phylum *Firmicutes* and *Bacteroidetes*. In which, the rise abundance of genus *Subdoligranulum* ($\beta$=0.410, *P* = 0.015), species *Bacteroides ovatus* ($\beta$=0.274, *P* = 0.025), family *Lachnospiraceae* ($\beta$=0.083, *P* = 0.017) and species *Eubacterium rectale* ($\beta$=0.077, *P* = 0.022) may contribute to a higher serum anti-mumps virus IgG. However, species *Ruminococcus callidus* ($\beta$=-0.063, *P* = 0.028) perhaps decrease it.

Moreover, several pathways including L-methionine biosynthesis (PWY-5345, $\beta$=0.464, *P* = 0.039), D-galactose degradation (PWY66-422, $\beta$=0.084, *P* = 0.042), purine nucleobases degradation (P164-PWY, $\beta$=0.073, *P* = 0.001), L-rhamnose degradation (RHAMCAT-PWY, $\beta$=0.072, *P* = 0.027), and pyrimidine ribonucleosides degradation (PWY-7209, $\beta$=0.065, *P* = 0.008) could up-regulate the concentrate of anti-mumps virus IgG in serum. On the contrary, a high capacity of several functional pathways like phospholipid biosynthesis in bacteria (PHOSLIPSYN-PWY, $\beta$=-0.315, *P* = 0.044), purine nucleotides de novo biosynthesis (PWY-841, $\beta$=-0.085, *P* = 0.008), L-arginine biosynthesis in *archaebacteria* (PWY-7400, $\beta$=-0.077, *P* = 0.025), and enterobacterial common antigen biosynthesis (ECASYN-PWY, $\beta$=-0.038, *P* = 0.020) played a role in lowering anti-mumps virus IgG. Since the pleiotropy of IVs presented, we selected MR-Egger regression method to access the causal relationships between *Subdoligranulum* (*P* = 0.022), *Bacteroides ovatus* (*P* = 0.029), L-methionine biosynthesis pathways (*P* = 0.046) and anti-mumps virus IgG.

#### Overlapping gut microbial features in the MR analyses

If we identify some common microbial traits that modulate different IgG levels, altering the abundance of specific microbial taxa or pathways was an excellent way to increase antiviral IgG levels simultaneously. Interestingly, ten certain microbial taxa or functional pathways were causally associated with multiple serum-specific antiviral IgG levels (Table 1). Both genus *Collinsella* and species *Bifidobacterium longum* were positively correlated with anti-IAV IgG and anti-measles virus IgG. The genus *Ruminococcaceae noname* was causally correlated with anti-measles virus IgG and anti-rubella virus IgG. In addition, seven pathways were positively or negatively associated with multiple anti-virus IgG levels, in which, a higher function capacity of purine nucleotides de novo biosynthesis (PWY-841) could decrease anti-measles virus IgG, anti-rubella virus IgG, and anti-mumps virus IgG levels at the same time. Moreover, the improvements in the L-alanine biosynthesis and nicotinamide adenine dinucleotide (NAD) salvage (PWY0-1061 and PYRIDNUCSAL-PWY) also could increase in these IgG levels.

As can be seen, the directions of associations of any microbial features with different IgG levels were consistent, contributing to the rise or fall of IgG levels, except a pathway of purine nucleobases degradation (P164-PWY). ​We suspected that the discordance may be caused by fewer IVs of this pathway and different choices of MR methods.

**Table 1:** Overlapping of forwarding causal associations between gut microbial features and antiviral IgG levels

Exposure	Outcome	Fixed-effect IVW	Random-effect IVW	Weighted median	MR-Egger	Heterogeneity		Pleiotropy
						*P* (IVW)	*P* (MR Egger)	*P* value
g_*Collinsella*	anti-IAV IgG	1.079(1.009,1.153)	1.079(0.997,1.167)	1.048(0.954,1.152)	1.082(0.639,1.835)	0.197	0.131	0.99
	anti-measles virus IgG	1.11(1.005,1.226)	1.11(1.067,1.155)	1.107(0.978,1.253)	0.969(0.521,1.801)	0.993	0.988	0.677
s_*Bifidobacterium_longum*	anti-IAV IgG	1.063(1.004,1.125)	1.063(1.013,1.116)	1.063(0.987,1.145)	1.015(0.774,1.33)	0.69	0.605	0.741
	anti-measles virus IgG	1.124(1.032,1.224)	1.124(1.019,1.24)	1.102(0.977,1.244)	1.269(0.781,2.063)	0.211	0.167	0.629
g_*Ruminococcaceae_noname*	anti-measles virus IgG	1.051(1.005,1.099)	1.051(1.019,1.084)	1.06(1,1.124)	1.081(0.852,1.372)	0.862	0.792	0.82
	anti-rubella virus IgG	1.069(1.001,1.142)	1.069(0.993,1.152)	1.034(0.944,1.133)	0.895(0.598,1.339)	0.258	0.245	0.412
PWY0-1061	anti-IAV IgG	1.062(1.003,1.125)	1.062(1.005,1.123)	1.073(0.996,1.156)	1.144(0.866,1.511)	0.462	0.381	0.615
	anti-measles virus IgG	1.103(1.013,1.201)	1.103(1.042,1.167)	1.1(0.984,1.23)	1.208(0.809,1.804)	0.874	0.82	0.666
PWY-6690	anti-IAV IgG	0.956(0.919,0.994)	0.956(0.927,0.986)	0.97(0.923,1.02)	1.085(0.891,1.322)	0.689	0.841	0.268
	anti-rubella virus IgG	0.909(0.83,0.995)	0.909(0.771,1.071)	0.973(0.838,1.13)	0.5(0.274,0.915)	0.01	0.125	0.142
PWY-841	anti-measles virus IgG	0.912(0.835,0.995)	0.912(0.838,0.992)	0.912(0.811,1.027)	0.633(0.428,0.938)	0.499	0.754	0.095
	anti-rubella virus IgG	0.808(0.708,0.922)	0.808(0.689,0.948)	0.813(0.674,0.981)	0.651(0.322,1.318)	0.159	0.13	0.554
	anti-mumps virus IgG	0.919(0.863,0.978)	0.919(0.869,0.972)	0.909(0.836,0.988)	1.034(0.779,1.371)	0.625	0.602	0.424
PYRIDNUCSAL-PWY	anti-measles virus IgG	1.087(1.005,1.176)	1.087(1.02,1.159)	1.114(1.002,1.238)	0.836(0.555,1.259)	0.748	0.831	0.236
	anti-rubella virus IgG	1.154(1.033,1.29)	1.154(1.048,1.272)	1.155(0.999,1.336)	1.713(0.873,3.36)	0.652	0.703	0.278
PWY-7446	anti-IAV IgG	0.952(0.919,0.986)	0.952(0.908,0.998)	0.937(0.89,0.985)	0.847(0.661,1.086)	0.101	0.108	0.39
	anti-mumps virus IgG	0.963(0.929,0.998)	0.963(0.939,0.988)	0.974(0.933,1.018)	1.1(0.921,1.313)	0.82	0.966	0.184
P164-PWY	anti-measles virus IgG	1.012(0.953,1.075)	1.012(0.949,1.079)	0.998(0.919,1.083)	0.75(0.59,0.952)	0.341	0.857	0.031
	anti-mumps virus IgG	1.076(1.031,1.123)	1.076(1.038,1.116)	1.049(0.99,1.111)	1.029(0.867,1.22)	0.716	0.656	0.607
PWY-HEME-BIOSYNTHESIS-II	anti-rubella virus IgG	0.867(0.777,0.968)	0.867(0.767,0.98)	0.831(0.713,0.969)	1.064(0.705,1.607)	0.267	0.275	0.335
	anti-mumps virus IgG	0.934(0.886,0.985)	0.934(0.882,0.99)	0.946(0.874,1.024)	0.886(0.716,1.096)	0.273	0.225	0.624

### Reverse MR analysis

Using anti-IAV IgG, anti-measles virus IgG, anti-rubella virus IgG, and anti-mumps virus IgG as exposure, we extracted 12, 11, 10, and six SNPs as IVs. The sum of values of PVE was 10.6%, 6.6%, 7.7%, and 5.1%. ​The *F* statistics of the IVs screened for the four IgG levels were all above 10, indicating that these IVs are strong instruments (Supplementary Table S7).

After reverse MR analyses, we found 24, 16, 20, and 10 gut microbial features affected by these four IgG levels respectively (Supplementary File: Figure S1 and S2) In addition, we identified five taxa (phylum *Verrucomicrobia*, species *Parabacteroides merdae*, *Alistipes finegoldii*, *Eubacterium eligens*, and *Bacteroides plebeius*) without any pathway that could be affected by more than one IgG levels (Table 2).

**Table 2:** Overlapping of potential reverse causal associations between antiviral IgG levels and gut microbial features

Exposure	Outcome	Fixed-effect IVW	Random-effect IVW	Weighted median	MR-Egger	Heterogeneity		Pleiotropy
						*P*(IVW)	*P* (MR Egger)	*P* value
anti-IAV IgG	p_*Verrucomicrobia*	0.727(0.534,0.989)	0.727(0.614,0.861)	0.685(0.472,0.993)	1.332(0.219,8.094)	0.975	0.972	0.523
anti-rubella virus IgG		1.159(1.001,1.343)	1.159(0.993,1.353)	1.215(0.986,1.498)	0.869(0.43,1.754)	0.355	0.326	0.437
anti-IAV IgG	s_*Parabacteroides_merdae*	1.385(1.028,1.865)	1.385(1.04,1.845)	1.189(0.802,1.763)	1.602(0.354,7.252)	0.505	0.415	0.851
anti-measles virus IgG		1.374(1.126,1.677)	1.374(1.059,1.783)	1.271(0.922,1.752)	1.683(0.625,4.535)	0.073	0.053	0.686
anti-IAV IgG	s_*Alistipes_finegoldii*	1.413(1.071,1.865)	1.413(1.071,1.864)	1.279(0.861,1.9)	0.678(0.169,2.724)	0.443	0.451	0.318
anti-mumps virus IgG		1.544(1.07,2.229)	1.544(1.112,2.145)	1.462(0.904,2.365)	2.169(0.215,21.854)	0.524	0.374	0.789
anti-rubella virus IgG	s_*Eubacterium_eligens*	1.205(1.045,1.389)	1.205(1.086,1.338)	1.167(0.968,1.407)	1.602(0.852,3.012)	0.829	0.837	0.395
anti-mumps virus IgG		1.523(1.043,2.225)	1.523(0.927,2.504)	1.193(0.684,2.081)	6.097(0.254,146.094)	0.142	0.138	0.45
anti-rubella virus IgG	s_*Bacteroides_plebeius*	0.757(0.576,0.995)	0.757(0.63,0.911)	0.742(0.526,1.047)	0.401(0.119,1.349)	0.888	0.925	0.327
anti-mumps virus IgG		2.111(1.023,4.356)	2.111(0.985,4.525)	2.99(1.087,8.229)	0.063(0.001,5.133)	0.351	0.59	0.211

## Discussion

This study performed comprehensive two-sample MR analyses to reveal the causal relationships between gut microbial features and antiviral IgG levels. Importantly, these inferred relationships were proved to be robust through various sensitivity analyses.

Previous studies have showed that some taxa influenced human immune system such as *Bacteroidetes* which could stimulate the innate immune system and *Ruminococcus* which contributed to upregulate immunity by producing short-chain fatty acid (SCFA), supporting the results of these taxa had positive effect on anti-IAV IgG in this study [37, 38]. In addition, we found that species *Desulfovibrio piger* may increase serum anti-measles virus IgG level. In fact, *Desulfovibrio piger* was the most common sulfate-reducing bacteria in healthy adults, and positively associated with beneficial genera like *Bacteroides* and *Ruminococcus* [39, 40]. For another, species *Coprococcus catus* had a negative impact on anti-measles virus IgG level, and it has proved to generate propanoic acid (a SCFAs) which had immunomodulatory properties [41, 42]. *Subdoligranulum* has been proved to simulate T_H_17 cell expansion and serum specific IgG [43]. *Bacteroides ovatus* was not only previously reported to modulate intestinal immunity, but also correlated with the host genetic variant [44, 45]. *Lachnospiraceae* of gut bacteria are abundant in healthy humans, and influence the hosts by producing SCFAs, converting bile acids, and facilitating colonization resistance against specific pathogens [46].

Interestingly, we also found ten microbial features could simultaneously regulate multiple antiviral IgG levels, such as genus *Collinsella*, *Ruminococcaceae noname*, and species *Bifidobacterium longum*, which will provide a reference for preventing different respiratory infectious diseases by adjusting same gut microbial traits. Previous studies have shown that *Collinsela* was one of the core microbiotas in healthy people, and a lower abundance of *Collinsela* predicted a higher respiratory infectious disease mortality [47]. *Bifidobacterium longum* has also attracted considerable attention among gut bacteria. In a double-blind study, *Bifidobacterium longum* stimulated immune function in 45 elderly hospitalized patients who had received influenza vaccines [48]. These surveys supported our findings of a high abundance of *Collinsela* and *Bifidobacterium longum* could enhance human immunity by elevating antibody concentrations. Moreover, this study confirmed that the functional capacity of microbial pathways, especially those involved in nucleotide and amino acid synthesis, may also affect the long-term immunity levels against respiratory infectious diseases.

The reverse MR analyses illuminated that human immune capacity also had an impact on the abundance of gut microbial features. However, the specific mechanism under antiviral antibodies regulate microbes was still unclear.

While our data-driven approach highlighted the potential of MR to uncover associations between microbiota and immune indicators, we should be cautious about the causalities. There were some limitations of this study that should be considered. First, similar to other MR studies, we have assumed linear relationships between gut microbial features and antiviral IgG levels in the MR model [49]. Nonetheless, we could not rule out the possibility that the relationships between gut microbial features and IgG levels were actually non-linear. Second, gender and age are the most common confounding factors in epidemiology, but we were unable to use GWAS summary data for stratified analyses to estimate and validate specific causal effects by gender or age stage. Besides, although MR design used genetic variants as IVs to minimize the effects of environmental confounding factors, it still cannot completely remove the confounding bias. Third, part of the microbial features in this study utilized few SNPs as IVs, resulting in a limited ability to identify causal relationships. Fourth, we performed FDR correction for *P* values, and used a nominal significance level of 0.05. If there are larger sample GWAS data available in the future, the relationship between gut microbiome and IgG level may become more significant after correction.

## Conclusions

This study has identified causal relationships between certain gut microbial features and serum-specific antiviral IgG levels. The results added new evidence for the influence of gut microbes on the human immune system, which provides a reference for enhancing population immunity to prevent respiratory infectious diseases after vaccination by adjusting gut microbes from a clinical and public health perspective.

### Electronic supplementary material

Below is the link to the electronic supplementary material.

Supplementary Material 1

Supplementary Material 2

## Data Availability

The summary statistics of 412 gut microbial features (Study accession numbers: GCST90027446-GCST90027857) and four anti-viral IgG level (Study accession numbers: GCST006350-GCST006353) used in this article could be downloaded from NHGRI-EBI GWAS Catalog (https://www.ebi.ac.uk/gwas/downloads/summary-statistics ). The main data generated or analyzed during this study are included in this published article/ Supplementary File, and Supplementary Tables, and further inquiries can be directed to the corresponding author.

## Abbreviations

IAV: Influenza A virus
IgG: Immunoglobulin G
IVW: Inverse variance weighted
WM: Weighted-median
GWAS: Genome-wide association study
MR: Mendelian randomization
IVs: Instrumental variables
DMP: Dutch Microbiome Project
MI: Milieu Interieur
S/CO: Signal/cut-off ratio
IA: Index Antibody
H^2^: Heritability
LD: Linkage disequilibrium
LDSC: Linkage disequilibrium score regression
MAF: Minimum allele frequency
CI: Confidence interval
MR-PRESSO: Mendelian Randomization Pleiotropy RESidual Sum analysis
PVE: Variance in phenotype explain
FDR: False discovery rate
