# Supplementary material for: Gut microbial features may influence antiviral IgG levels after vaccination against viral respiratory infectious diseases: the evidence from two-sample bidirectional mendelian randomization
Source: BMC Infect Dis. 2024 Apr 23;24:431. doi: 10.1186/s12879-024-09189-0 (PMC11036767; doi:10.1186/s12879-024-09189-0)
Supplement: Supplementary file 1 — Supplementary Material 1 [file 12879_2024_9189_MOESM1_ESM.docx]

Supplementary File


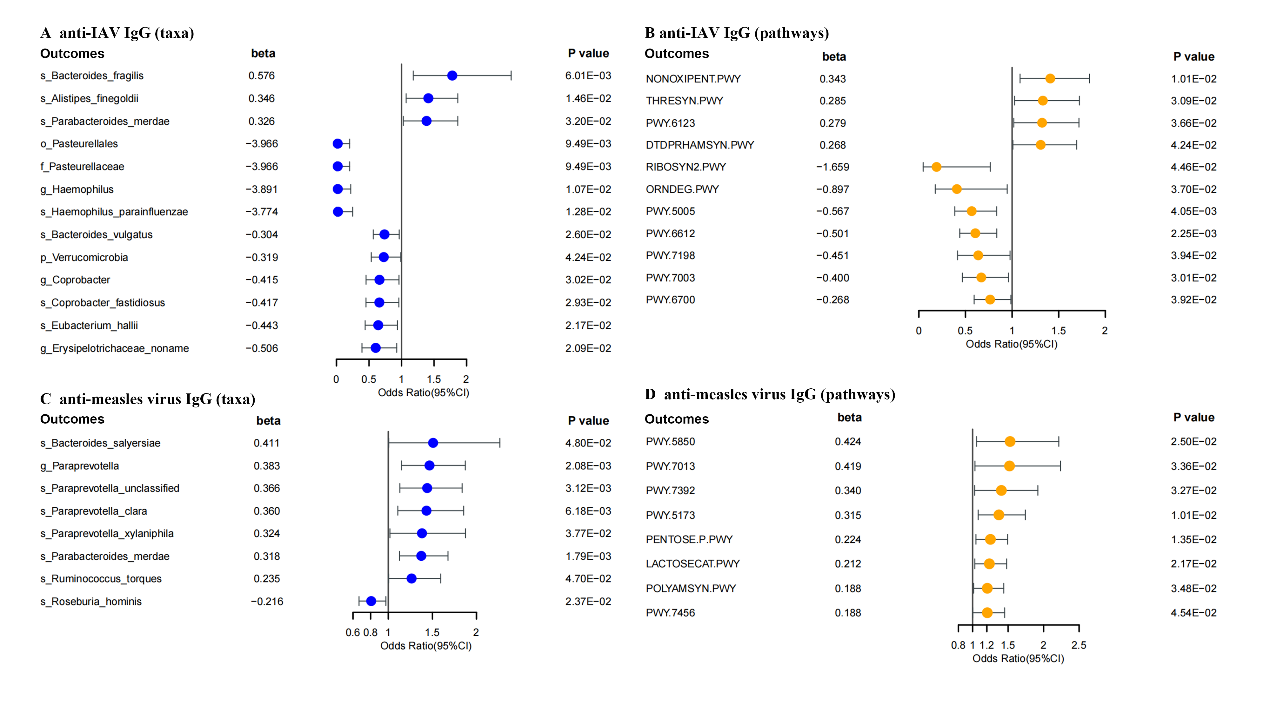


Figure S1. Causal effects of anti-IAV IgG and anti-measles virus IgG levels on gut microbial features. The forest plot represents the MR estimates beta and 95%CI values of the odds ratio of different anti-IAV IgG levels (A, B) and anti-measles virus IgG levels (C, D) on gut microbial features. The features are grouped into taxa (color in blue) and pathways (color in orange) and arranged according to the effect value.


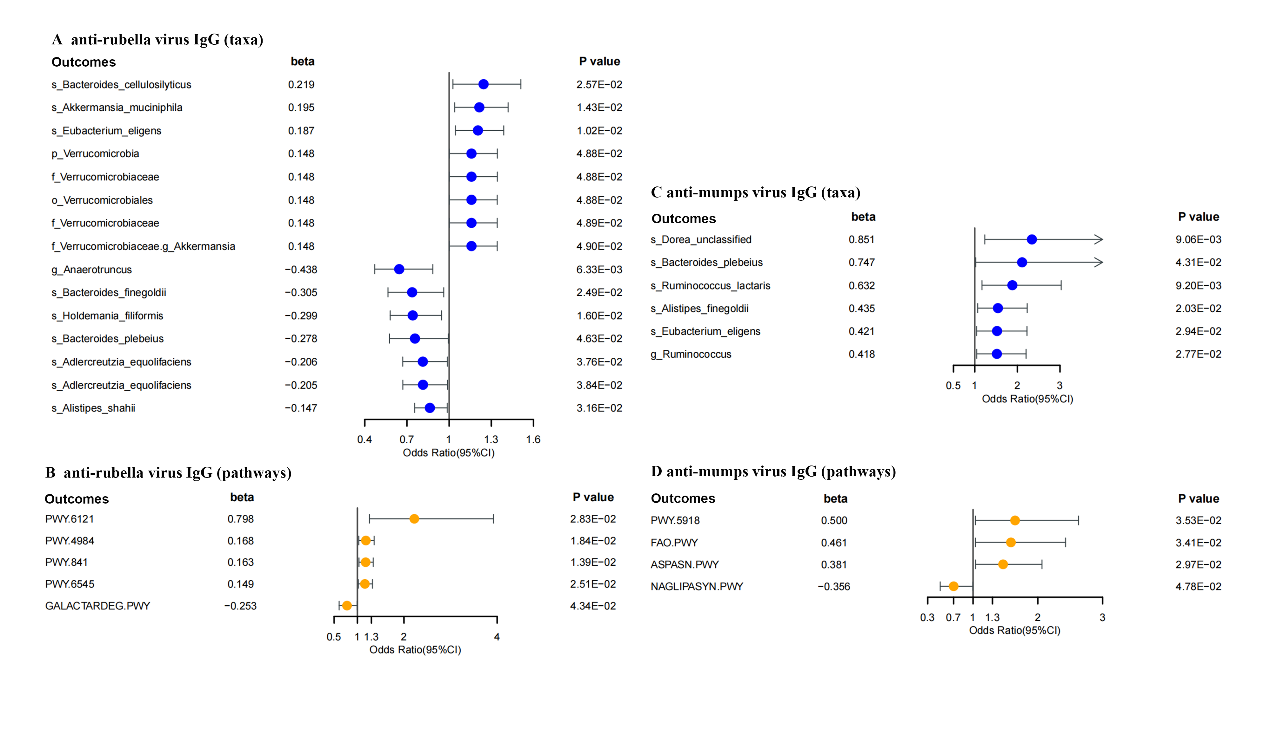


Figure S2. Causal effects of anti-rubella virus IgG and anti-mumps virus IgG levels on gut microbial features. The forest plot represents the MR estimates beta and 95%CI values of the OR of different anti-rubella virus IgG levels (A, B) and anti-mumps virus IgG levels (C, D) on gut microbial features.
